# Relationship Between Active Aging and Perceived Aspects of Home Among Persons Aged 55+ Listed With an Interest in Relocation

**DOI:** 10.1177/30495334251366584

**Published:** 2025-08-30

**Authors:** Frida Nordeström, Björn Slaug, Magnus Zingmark, Marianne Granbom, Susanne Iwarsson

**Affiliations:** 1 Lund University, Sweden; 2 Östersund Municipality, Sweden; 3 Umeå University, Sweden

**Keywords:** older adults, aged, home environment, healthy aging

## Abstract

The objectives of this study were to explore active aging among people listed with an interest in relocation, and the relationships between active aging and aspects of perceived home. Using cross-sectional data from the 2022 RELOC-AGE project (*N* = 1,509, mean age = 70 years), linear regression analysis was conducted. Women and individuals with higher self-rated health and education reported higher levels of active aging. After adjusting for confounders, Housing Satisfaction (decrease in Housing satisfaction led to lower active aging scores (β = −17.8, 95% CI [−28.6, −7.0] for neither satisfied nor dissatisfied), and Meaning of home relationship (β = 4.6, 95% CI [3.4, 5.8]), was positively associated with active aging, “Housing-Related Control Beliefs” showed a significant negative relationship (β = −14.6, 95% CI [−17.1, −12.1]). These findings are significant for promoting health and well-being among older adults. They add knowledge about home as a key factor for active aging and could be valuable for policymakers, housing authorities, and healthcare and social services staff involved in aging and housing issues.

## Introduction

Active aging has become a central focus in policies developed by the World Health Organization (WHO, 2002) and the European Union (EU; European Commission, 2012). The determinants of active aging (WHO, 2002) encompass economic, social, personal, physical, behavioral, health, and social service factors, which vary across individuals and regions. Inequalities in these determinants are reflected in reports that used tools like the Active Aging Index (AAI) to monitor demographic trends and inform policy discussions ((UNECE, 2013, 2018). However, there are few studies that explored differences in active aging regarding basic factors such as sex, educational level and health. On the individual level, active aging is a personal process of making choices and engaging in activities that help improve one’s well-being (Rantanen et al., 2019). Such activities should align with personal goals, possibilities, and opportunities. Research has revealed links between active aging, Quality of Life, Life-Space Mobility (Rantanen et al., 2021), and health literacy (Eronen et al., 2021). These results enhance the understanding of the complex nature of aging well and underscore the importance of holistic approaches to promote health and well-being in later life. This study considers active aging in the context of housing, which hitherto has scarcely been addressed in research.

The home is an essential determinant of health, as mastery of one’s life and effective coping with daily demands are prerequisites for an independent and autonomous life (Baltes et al., 1998). The relationship between home and health is multifaceted, and influences factors such as residential decision-making and access to services, particularly among very old adults (Iwarsson et al., 2016). Thus, recognizing the significance of the home in promoting independence and autonomy is crucial for fostering active aging and overall well-being. The determining factors for overall satisfaction in the home have been found to be the perceived rather than objective factors (Amián et al., 2021).

A model of perceived home was developed to acknowledge individual emotions and preferences in terms of housing, encompassing distinctive emotional attributes rooted in individual preferences and sentiments that evolve over time (Oswald et al., 2006). This model comprises four domains: Housing Satisfaction, Usability of Home, Meaning of Home, and Housing-Related Control Beliefs. Housing Satisfaction captures perceived qualities of the home, focusing on an evaluation of the relationship between the person and his/her home environment. The usability aspect stems from occupational theory and the ecological theory of aging (Lawton & Nahemow, 1973), and captures the perceived usability of the housing environment for performing daily activities (Oswald et al., 2006). Meaning of Home describes the accumulation of place attachment when people form affective, cognitive, behavioral, and social bonds to a particular setting, thereby transforming a space into a place. Housing-Related Control Beliefs take striving for control in the home into account and explain events at home as contingent upon either one’s own behavior or luck, chance, fate, and powerful others. Overall, perceived home deals with unique emotional qualities based on personal preferences and feelings that develop over time.

Studies that examine how older adults perceive their homes have found that for adults aged 75+ or 85+ in Europe, living in a home with better accessibility and having control over the home situation is linked to a higher level of independence in daily activities and an improved sense of well-being (Oswald, Wahl, Schilling, & Iwarsson, 2007). Older adults with a higher degree of disability tend to perceive their homes as less usable and meaningful, and they perceive less control over their home situation (Tomsone et al., 2013; Wahl et al., 2009). Perceived home plays a role for health and well-being among younger older adults as well. In two studies using the same sample of 67 to 70-year-olds (Haak et al., 2015; Kylén et al., 2017), the relationships between perceived home and several symptoms were explored. The findings suggest that reporting fewer symptoms is associated with a higher Meaning of Home, and more control over the home situation (Haak et al., 2015). Depression was less common where there was a cognitive–emotional bond (i.e., meaning of home) and perceived control in the home (Kylén et al., 2017). Oswald et al.’s model (Oswald et al., 2006) outlines the multifaceted nature of perceived home, encompassing emotional attributes shaped by individual preferences and evolving sentiments. However, it raises questions about the extent to which perceived aspects of home can capture the complexities of individual experiences and social dynamics within the home environment.

To the best of our knowledge, only two studies have examined active aging in the context of housing. The first study compared the levels of active aging among senior housing residents and community-dwelling older adults in Finland (Siltanen et al., 2024). This study showed that despite the social and supporting environment, senior housing residents’ possibilities for active aging seem to be compromised, potentially leading to unmet activity needs. The second study revealed that perceived home moderates the relationship between functional limitations and active aging (Slaug et al., 2024). These results suggest that a home imbued with personal meaning fosters engagement in meaningful activities, thereby supporting active aging even in the presence of functional limitations. This underscores the importance of not only the physical housing environment but also the social and cultural aspects of home for active aging.

A significant knowledge gap remains regarding the interplay between active aging and perceived home dynamics. And, when it comes to home and health issues regarding older adults, previous literature has primarily addressed very old or frail old adults (Roy et al., 2018; Sixsmith et al., 2014; Zimmermann et al., 2021). This underscores the need for research to enhance the understanding of the relationship between active aging and perceived home across various age groups (WHO, 2018).

The aim of this study was twofold: (1) to examine differences in active aging regarding age, sex, educational level, and health, and (2) to explore relationships between active aging and aspects of perceived home.

## Material and Methods

### Study Context

The RELOC-AGE research program rests on the notion of proactive aging (Iwarsson et al., 2023), thus targeting people at an early phase of the aging process to generate knowledge based on a health promotion perspective, following housing and health trajectories over time. Consequently, participants were included already from age 55. For this study we used data from the Prospective RELOC-AGE project, registered at ClinicalTrials.gov [NCT04765696] (ClinicalTrials.gov, 2021). See study protocol (Zingmark et al., 2021); International Registered Report Identifier (IRRID): DERR1-10.2196/31137.

Prospective RELOC-AGE was approved by the Swedish Ethical Review Authority (Nos. 2020-03457, 2022-01287-02). The present study adhered to the STROBE guidelines for reporting observational research, see Appendix 1.

### Participants

To recruit an information rich sample with respect to relocations, the study population consisted of persons aged 55 years or older with a postal address in Sweden and listed with an interest in relocation at one of three housing companies: two municipal public housing companies and one national provider of tenant-owned dwellings. Those with severe cognitive impairments or insufficient language skills were excluded. At baseline in 2021, 1,964 individuals participated. For this study, we analyzed cross-sectional data from the 1-year follow-up (2022), which included 1,509 participants (77% response rate). Although no formal power calculation was performed, the large sample allowed for robust statistical analysis.

At baseline, after having received information about the study, informed consent for the survey study was obtained by requiring participants to check a consent box after login to the web portal used for the data collection, before proceeding with the online questionnaire. This ensured that participants acknowledged their rights and agreed to participate voluntarily. More than half of the sample were women (55.7%); mean age 70 years (*SD* = 7.6). More than two-thirds had university education (68%), 62% were married/registered partners, 80% owned their dwelling, and 56% rated their health as excellent or very good (see Table 1).

**Table 1.** Participant Characteristics, *N* = 1,509.

Variable	Mean (SD), or *n* (%)	Missing responses
Age	70 (7.6)	0
Sex		0
Women	840 (55.7)	
Educational level		7
Compulsory (9 years)	114 (7.6)	
Upper secondary (2 years)	133 (8.8)	
Upper secondary (3–4 years)	230 (15.2)	
University (less than 3 years)	320 (21.2)	
University (3 years or more)	705 (46.7)	
Marital status		8
Married/registered partner	930 (61.6)	
Unmarried	219 (14.5)	
Widowed	144 (9.5)	
Divorced	208 (13.8)	
Self-rated health		4
Poor	23 (1.5)	
Fair	217 (14.4)	
Good	418 (27.7)	
Very good	562 (37.2)	
Excellent	285 (18.9)	
Dwelling type		6
Apartment	801 (53.1)	
Single-family house	694 (46.0)	
Dwelling ownership	1,211 (80.3)	30
Type of residential area		20
Urban	936 (62.0)	
Semi-urban	400 (26.5)	
Rural	153 (10.1)	
Household composition		14
Single person household	457 (30.3)	
Family-based household^a^	1,038 (68.2)	

a Includes living with a spouse, a child, a sibling, a parent, or another adult.

### Instruments

#### Active Aging

Active aging was assessed using the University of Jyväskylä Active Aging Scale (UJACAS), a self-assessment scale developed to understand active aging as an individual pursuit applicable regardless of health conditions (Rantanen et al., 2019). UJACAS encompasses multiple dimensions of active aging such as physical, social, and cognitive engagement. We used active aging as the dependent variable, represented by a total score (range 0–272) based on four sub-scales (Goals, Ability, Opportunity, and Activity), with higher scores indicating more active aging. UJACAS consists of 17 items, each rated on the four sub-scales with scores ranging from 0 (least active) to 4 (most active). In this study, we calculated sub-scale scores (range 0–68) and composite total score (0–272) based on sub-scale scores. Cronbach’s alpha for the subscales and total scale were .8 to .9 (Hobart & Cano, 2009; Streiner et al., 2015). Missing data for sub-scale scores were imputed, allowing a maximum of two missing items per sub-scale and eight for the total score.

#### Perceived Home

##### Housing Satisfaction

Housing Satisfaction encompasses an overall evaluation of the home’s condition using the single question: “Are you satisfied with your dwelling?” (scale 1–5; higher score = greater satisfaction; Oswald et al., 2006). To enhance residual distribution and adherence to homoscedasticity assumptions, response options 1 and 2 were merged.

##### Usability

The Usability in My Home (UIMH) instrument pertains to the individual’s subjective assessment of how supportive or restrictive the home environment is in facilitating activity performance (Fänge & Iwarsson, 2005b). To lessen participant burden, usability was measured using four UIMH items targeting the activity components (Fänge & Iwarsson, 2003). The respondents rated the extent to which they perceived the design of the home to be usable (scale 1–5) for managing personal hygiene, dressing, and bathroom visits; cooking or heating food; dishwashing, cleaning, and flower care; and laundry and other clothing care. A mean score was calculated, with a higher score indicating better/more usability.

##### Meaning of Home

The Meaning of Home questionnaire evaluates the subjective meanings people associate with their homes across four dimensions: physical, behavioral, cognitive/emotional, and social (Boonyaratana et al., 2021; Oswald et al., 2006). It comprises 28 statements (rated from 0 to 10) categorized into four sub-scales: Behavioral (six items), such as managing tasks independently; Physical, (seven items), such as residing in a well-designed space tailored to personal needs; Cognitive/Emotional (10 items), such as feeling secure; and Social (five items), such as being able to host visitors. For eight negatively phrased statements the responses were reversed before calculating a mean total scale score (higher = stronger Meaning of Home).

##### Housing-Related Control Beliefs

Housing-Related Control Beliefs entail the notion that housing events are influenced by a person’s own behavior, or factors outside personal control (Oswald et al., 2006). Because the subscale Internal Control has low construct validity (Boonyaratana et al., 2021), only the sub-scales of external control (Powerful Others; Chance) were used (Oswald et al., 2003). The 16 items were rated on a five-point scale (rang 1–5). The item scores were used to calculate a mean score; lower scores = higher perceived control.

Cronbach’s alpha for the usability, Meaning of home, and Housing-Related Control Beliefs were .8 respectively (Hobart & Cano, 2009; Streiner et al., 2015).

##### Confounders

Confounders (age, sex, educational level, self-rated health) were chosen based on previous research (Ekström et al., 2016; Kylén et al., 2017; Oswald, Wahl, Schilling, Nygren, et al., 2007; Rantanen et al., 2021). Age and sex were based on personal identity number information, while all other variables were self-reported. Age was categorized into four groups (55–64, 65–74, 75–84, and 85–94) analyzing differences in active aging, and treated as a continuous variable when examining the relationship between active aging and perceived home.

### Data Analyses

One-way ANOVA was conducted to test for differences in UJACAS subscale and total scale score between age groups, sex, education, and self-rated health. Following post-hoc analyses, Bonferroni adjustment was applied to reduce the risk of Type I errors.

Linear regressions (ordinary least square, OLS) were used to explore the relationship between the dependent variable active aging total score and the four perceived home aspects. Initially, separate univariable analyses were conducted for each perceived home variable, followed by a multivariable analysis incorporating all perceived home variables. A second multivariable analysis was conducted, adjusting for confounding variables as described above. For ordinal variables, the most positive value was used as a reference value, as higher education and self-rated health were more likely to be associated with a higher level of active aging. Multicollinearity was investigated between the four aspects of perceived home using Spearman’s rho, and was found to be negligible. Model validation was performed by visual inspection of residuals.

*p*-Values < .05 were used to define statistical significance; all tests were two-sided. Analyses were performed using IBM SPSS Statistics for Windows, Version 28.0.

## Results

### Active Aging

The Ability sub-scale received the highest mean rating (63.4), while Activity received the lowest (43.9) (Table 2). Women rated their active aging as significantly higher than men on the sub-scales Goals and Activity, and on the total scale (*p* < .001, .004, and <.001). Post-hoc testing revealed that there were numerous pairwise significant differences, for example, between 55 and 64 year olds and 75 to 84 year olds, and between 65 and 74 and 75 to 84 year olds (subscale Goals, Ability, Opportunity and Total scale). Also between educational level Compulsory school and University 3 years or more across subscales and total scale, and self-rated health Fair and Good, and Excellent across subscales and total scale (see Appendix 2).

**Table 2.** Distribution and Differences of Active Aging, Sub-Scales and Total Score, *N* = 1,509.

	Subscale range 0–68				
	Goals	Ability	Opportunity	Activity	Total score range 0–272
Variable	Mean (standard deviation)				
UJACAS score					
Total sample (*N* = 1,509)	48.6 (8.8)	63.4 (6.7)	58.5 (9.2)	43.9 (9.5)	214.4 (27.3)
Missing	11	11	11	11	28
Age group, years					
55–64 (*n* = 421)	48.9 (9.0)	64.5 (5.7)	59.6 (8.9)	42.9 (9.9)	216.0 (25.4)
Missing	1	4	0	2	0
65–74 (*n* = 675)	49.7 (8.5)	63.9 (6.4)	59.4 (8.4)	45.0 (9.2)	218.0 (26.0)
Missing	6	4	7	5	13
75–84 (*n* = 380)	46.9 (8.4)	62.1 (6.9)	56.2 (9.5)	43.4 (9.5)	208.6 (28.0)
Missing	4	3	2	3	8
85–94 (*n* = 33)	44.9 (11.0)	54.9 (12.0)	49.1 (13.9)	40.6 (10.3)	189.4 (42.3)
Missing	0	0	1	1	1
* p*-value	<.001	<.001	<.001	<.001	<.001
Sex					
Women (*n* = 834)	50.0 (8.5)	63.5 (6.8)	58.4 (9.6)	44.5 (8.5)	216.6 (27.7)
Missing	6	5	4	6	17
Men (*n* = 664)	46.9 (8.7)	63.3 (6.5)	58.5 (8.8)	43.1 (9.5)	211.8 (26.5)
Missing	5	6	7	5	11
* p*-Value	<.001	.5	.9	.004	<.001
Educational level					
Compulsory (9 years) (*n* = 114)	44.1 (8.8)	61.7 (7.7)	55.3 (10.0)	41.8 (9.1)	202.8 (30.3)
Missing	0	0	0	0	0
Upper secondary (2 years) (*n* = 132)	46.2 (8.2)	62.1 (7.0)	57.6 (8.4)	41.9 (9.1)	207.69 (25.8)
Missing	1	2	1	1	3
Upper secondary (3–4 years) (*n* = 229)	47.9 (8.5)	63.6 (6.1)	58.2 (9.2)	43.2 (9.6)	213 (26.3)
Missing	1	3	2	0	4
University (<3 years) (*n* = 315)	49.1 (8.6)	63.6 (6.8)	58.5 (9.3)	44.4 (9.7)	215.5 (27.5)
Missing	5	2	4	4	9
University (3+ years) (*n* = 701)	49.9 (8.6)	63.9 (6.3)	59.3 (9.0)	44.6 (9.5)	217.8 (25.9)
Missing	4	4	4	6	12
* p*-Value	<.001	.002	<.001	.002	<.001
Self-rated health					
Poor (*n* = 23)	43.9 (10.8)	42.6 (12.6)	36.7 (13.8)	30.5 (9.6)	153.9 (39.5)
Missing	0	0	0	0	0
Fair (*n* = 217)	46.0 (8.8)	56.9 (8.3)	50.0 (10.0)	39.5 (9.6)	192.4 (29.0)
Missing	2	2	2	1	4
Good (*n* = 418)	47.0 (8.5)	62.9 (5.6)	57.1 (8.2)	43.0 (8.9)	209.8 (23.7)
Missing	5	4	5	5	12
Very good (*n* = 562)	49.6 (8.4)	65.6 (3.9)	61.1 (6.9)	44.9 (9.1)	221.3 (21.6)
Missing	4	3	3	3	8
Excellent (*n* = 285)	51.7 (8.2)	66.5 (3.0)	63.6 (5.5)	47.7 (8.8)	229.6 (19.7)
Missing	0	2	1	2	4
* p*-value	<.001	<.001	<.001	<.001	<.001

*Note.* Higher scores indicate higher active aging (Rantanen et al., 2019). For results from Bonferroni-corrected post-hoc analyses, see Appendix 2.

### Relationship Between Active Aging and Perceived Home

The mean scores (SD) for Housing Satisfaction, usability, Meaning of Home, and Housing-Related Control Beliefs were 4.7 (0.7), 4.8 (0.4), 8.0 (1.0), and 1.8 (0.5) respectively. All univariable analyses were statistically significant (*p* < .001). Higher Housing-Related Control Beliefs showed a negative relationship with active aging, while higher Housing Satisfaction, usability, and Meaning of Home showed positive relationships (Table 3).

**Table 3.** Relationships Between Active Aging and Perceived Home, *N* = 1,509.

	Regression model		
Variable	Univariable	Multivariable	Adjusted multivariable with confounders
*Independent variable*		β [95% confidence interval]	
Housing satisfaction (ref. very satisfied)			
Not satisfied	−18.1 [11.4, 24.9]***	−7.0 [0.6, 13.3]	−4.3 [−10.2, 1.6]
Neither satisfied nor unsatisfied	−40.9 [28.5, 53.3]***	−13.6 [1.9, 25.3]	−17.8 [−28.6, −7.0]***
Somewhat satisfied	−9.8 [6.4, 13.2]***	−2.3 [−1.0, 5.6]	−2.0 [−5.1, 1.1]
Usability	12.8 [9.7, 16.0]***	1.1 [−2.1, 4.4]	−0.8 [−3.8, 2.2]
Meaning of home	9.0 [7.7, 10.3]***	5.3 [4.1, 6.6]***	4.6 [3.4, 5.8]***
Housing-related control beliefs	−24.9 [−27.3, −22.6]***	−21.1 [−23.6, −18.7]***	−14.6 [−17.1, −12.1]***
*Confounders*			
Age			0.2 [0.1, 0.4]**
Men (ref. women)			−2.1 [4.4, −0.3]
Educational level (ref. university 3 years or longer)			
Compulsory			−7.7 [−12.3, −3.2]*
Upper secondary 2 years			−6.5 [−10.6, −2.3]**
Upper secondary 3–4 years			−4.1 [−7.4, −0.8]**
University up to 3 years			−1.5 [−4.4, 1.5]
Self-rated health (ref. excellent)			
Poor			−49.1 [−59.2, −39.0]*
Fair			−26.6 [−30.8, −22.3]*
Good			−13.3 [−16.8, −9.8]*
Very good			−5.0 [−8.2, 1.8]*
Intercept [95% CI]		204.4 [185.0, 223.7]***	205.4 [185.9, 224.9]***
No. of observations		1,379	1,371
*R* ^2^		.29	.40

Note. Ordinary least square (OLS) used as estimator; ***p ≤ .001, **≤.01, *≤.04. VIF-parameters multivariable 1.1–1.3; multivariable with confounders 1.1–1.4.

In the multivariable analysis, the significant relationships remained positive for Meaning of Home and negative or Housing-Related Control Beliefs, and the relationships remained stable in the adjusted model (*p* < .001; 40% explained variance, *R*^2^).

## Discussion

This study produced knowledge about active aging and perceived aspects of home. The main finding is the relationship between active aging and meaning of home and housing-related control beliefs. One plausible explanation for this relationship could be that individuals who perceive their home as meaningful and have a sense of control over their home situation are more likely to engage in activities that are conducive to active aging. The correlation between higher educational level, better self-rated health, and increased engagement in active aging aligns with what could be anticipated.

The housing satisfaction variable shows a positive relationship with active aging, but only the category “very satisfied” remained consistently significant across all models. In the multivariable analysis including confounding variables, all levels of housing satisfaction were positively associated with active aging. Notably, the category indicating neither satisfaction nor dissatisfaction exhibited the most significant impact, which could imply a potential S-shaped relationship. This might be partly explained by a housing or residential satisfaction paradox, describing that it is possible to be both content and discontent about the home at the same time. This is because for some individuals, housing satisfaction is more about social belonging than the attributes of the dwelling itself (Wahl & Oswald, 2010). Previous research also indicates that there is no discernible difference in housing satisfaction among older adults, regardless of variances in dependence in ADL (Tomsone et al., 2013). While the present study sheds new light on such a dynamic, more research is needed to further increase our understanding of the mechanisms driving the relationship.

For usability, the association diminished when other variables were considered and the relationship with active aging decreased substantially, indicating that the initial association was not robust when adjusted for confounders. Alternatively, the association could be influenced by the high mean for usability observed in the sample. Previous research has indicated that the questions used to capture usability may not be sufficiently sensitive to capture variation in a sample (Fänge & Iwarsson, 2005a, 2005b).

The relationship between meaning of home and housing-related control beliefs appears consistent, as indicated by the coefficients and confidence intervals across models. Research indicates that fewer symptoms are tied to higher meaning of home and lower housing-related control, but not usability (Haak et al., 2015). Tomsone et al. (2013) found that perceived usefulness and meaningfulness in the home were lower in ADL-dependent groups, and housing control was reported as lower among very old adults. Higher housing-related control was linked to greater purpose in life (Kylén et al., 2017). Very old adults who perceived their home useful, meaningful, and housing control were more independent in daily activities, reported better well-being, and less depressive symptoms (Oswald, Wahl, Schilling, Nygren, et al., 2007). As overall control beliefs are important for health and well-being as we age (Lachman et al., 2011), such findings suggest that older adults who find meaning in their home and have a greater sense of control are more likely to exhibit active aging regardless of age. When studying active aging and perceived home, Slaug et al. (2024) found that perceived home moderates the negative association between functional limitations and active aging. Specifically, higher levels of usability and meaning of home help mitigate the negative impact of functional limitations, supporting active aging despite these limitations. Accordingly, the current study contributes additional knowledge about dynamics involving housing satisfaction, usability, meaning of home, and functional limitations and associations with active aging. Thus, considering the results of our study, interventions aimed at promoting active aging should consider multiple factors, including perceived aspects of home. While current rehabilitation interventions—such as housing adaptations and provision of assistive devices—remain relevant for people with functional limitations, these are examples of reactive rather than proactive approaches (Iwarsson et al., 2023). Transitioning from disease management to health promotion is crucial. Interventions that promote strategic decision-making and adaptability are proactive measures, supporting this transition and improving housing interventions for older adults. Such interventions should enhance satisfaction, meaningfulness, and a sense of control in the home environment, and could significantly contribute to the well-being of older adults across different age groups.

The finding that younger older adults rate their active aging higher was expected, and is consistent with previous research (Hsu et al., 2019; Nyqvist et al., 2022). Our findings, indicating that women generally report higher levels of active aging compared to men, align with Siltanen et al. (2024). They investigated active aging among individuals in both ordinary and senior housing, categorized by sex, and found that women rated their overall active aging higher in both types of housing. However, in our study, the difference between men and women did not surpass the standard error of measurement (SEM; Nordeström et al., 2024), which could indicate that, although statistically significant, it may not represent meaningful differences. Additionally, differences in the subscale scores between our study and the Finnish study may be attributed to sample differences and underscore the importance of considering specific contexts and populations when interpreting and comparing research findings on active aging. Investigating active aging on the societal level, women generally fare worse than men in almost all countries, including the Nordic region (UNECE, 2013, 2018). This could suggest that the AAI may reflect underlying societal norms and structures that disadvantage women in certain areas. For instance, addressing the economic factor in the index, women may face barriers in accessing employment opportunities and have lower overall lifetime earnings, which leads to Swedish women in general having lower total pension payouts than men (Inspektionen för socialförsäkringen [ISF], 2017). While one item in the UJACAS self-assessment addresses financial stability, the assessment also encompasses Goals, Opportunity, Ability, and Activity, providing a comprehensive understanding of individuals’ efforts to enhance their well-being. This underscores the multidimensional nature of active aging and emphasizes the importance of considering diverse aspects of well-being. Future studies in the field would benefit from exploring potential differences between men and women and assessing the extent to which different measurement methods accurately and equitably capture these differences.

While our results indicate that those with higher levels of education and better self-rated health rate their active aging as higher, significant differences remained for education after applying the Bonferroni adjustment, particularly in the Goals, Ability, and Opportunity subscales. For self-rated health, the impact on Ability, Opportunity, and Activity remained stronger, aligning with previous findings from Rantanen et al. (2021). Given previous research suggesting a link between health literacy and active aging (Eronen et al., 2021), and the established relationship between educational level and health literacy (Aljassim & Ostini, 2020; Svendsen et al., 2020), it would be prudent to investigate further the potential mediating role of health literacy in the relationship between education and active aging.

### Methodological Considerations

The large sample size in this study allowed for robust statistical analysis, enhancing the generalizability of the findings to the population of adults aged 55+ and considering relocation. While not common in research on aging, including individuals as young as 55 years old is essential for studies on housing and health based on a proactive approach (Iwarsson et al., 2023).

Considering the longitudinal ambition of Prospective RELOC-AGE, applying the inclusion criterion “interested in relocation” was necessary to attain sufficient variance in the sample when it comes to actual prospective relocations. As outlined in the Prospective RELOC-AGE study protocol (Zingmark et al., 2021), the recruitment strategy was aimed at including information-rich participants rather than ensuring representativeness, consistent with the exploratory and mixed methods design of the overall program. However, as we did not study relocations in the present study, and reflecting critically on this inclusion criterion it may introduce selection bias. Individuals who are contemplating relocation may be more likely to experience dissatisfaction with their current housing, be more ready for change of their situation, or maybe place higher value on certain housing-related factors compared to those without any interest to move to another dwelling. While such unknown factors could affect the observed relationships between active aging and perceived aspects of home, the explorative studies from Prospective RELOC-AGE contributes important knowledge for future research.

Moreover, it is important to note that we lack comprehensive data on the total population of individuals aged 55+ in Sweden who are listed with an interest in relocation. Comparing our sample to the general population aged 55+ in Sweden, we observed that a large proportion of our participants were highly educated and reported good health (Statistics Sweden, 2022). Thus, the sample is skewed toward a more favorable status in terms of sociodemographic status and health, and the results should be interpreted with this in mind.

The cross-sectional design of the present study is a limitation, preventing us from determining the causality or direction of the relationship between active aging and perceived home. Longitudinal follow-up data collections that will be used for studies to confirm the direction of relationships over an extended period are underway.

## Conclusion

The present study sheds light on the insufficiently studied dynamic interplay between active aging and perceived aspects of home among adults aged 55+ listed with an interest in relocation. The value for further research lies in deepening our understanding of meaning of home and housing-related control beliefs to promote active aging. This insight can be valuable for targeted measures aimed at enhancing the well-being of older adults by optimizing their home. Furthermore, the results point to a need to investigate potential moderating factors such as socio-economic status, cultural factors, or individual personality traits. The results highlight variations in associations with active aging, particularly in terms of self-rated health. This information can be valuable for policymakers, housing authorities, and professionals in healthcare and social services involved in issues related to aging and housing. It suggests that initiatives focused on enhancing education and health outcomes might lead to more positive views of active aging, benefiting not only individuals but also society at large.

## Appendix

**Appendix 1:** STROBE Checklist for the Cross-Sectional Study “Relationships Between Active Aging and Perceived Aspects of Home” (Nordeström et al., 2024).

	Item no.	Recommendation	Page no.	Relevant text from manuscript
Title and abstract	1	(*a*) Indicate the study’s design with a commonly used term in the title or the abstract	1	
		(*b*) Provide in the abstract an informative and balanced summary of what was done and what was found	2	
*Introduction*				
Background/rationale	2	Explain the scientific background and rationale for the investigation being reported	3–6	
Objectives	3	State specific objectives, including any prespecified hypotheses	6	
*Methods*				
Study design	4	Present key elements of study design early in the paper	7	
Setting	5	Describe the setting, locations, and relevant dates, including periods of recruitment, exposure, follow-up, and data collection	6–7	
Participants	6	(*a*) *Cross-sectional study*—Give the eligibility criteria, and the sources and methods of selection of participants	6–7	
Variables	7	Clearly define all outcomes, exposures, predictors, potential confounders, and effect modifiers. Give diagnostic criteria, if applicable	10–12	
Data sources/ measurement	8*	For each variable of interest, give sources of data and details of methods of assessment (measurement). Describe comparability of assessment methods if there is more than one group	10–12	
Bias	9	Describe any efforts to address potential sources of bias	29–30	
Study size	10	Explain how the study size was arrived at	30	
Quantitative variables	11	Explain how quantitative variables were handled in the analyses. If applicable, describe which groupings were chosen and why	10–12	
Statistical methods	12	(*a*) Describe all statistical methods, including those used to control for confounding	13	
		(*c*) Explain how missing data were addressed	10	
*Results*				
Participants	13*	(a) Report numbers of individuals at each stage of study—eg numbers potentially eligible, examined for eligibility, confirmed eligible, included in the study, completing follow-up, and analysed	7	
Descriptive data	14*	(a) Give characteristics of study participants (eg demographic, clinical, social) and information on exposures and potential confounders	7–9	Table 1
		(b) Indicate number of participants with missing data for each variable of interest	7–9, 16–21 & 23–25	Table 1, 2, 3
Outcome data	15*	*Cross-sectional study—*Report numbers of outcome events or summary measures	13–21	Table 2
Main results	16	(*a*) Give unadjusted estimates and, if applicable, confounder-adjusted estimates and their precision (e.g., 95% confidence interval). Make clear which confounders were adjusted for and why they were included	16–21 & 23–25	Table 3
		(*b*) Report category boundaries when continuous variables were categorized	12	
Other analyses	17	Report other analyses done—eg analyses of subgroups and interactions, and sensitivity analyses	13	
*Discussion*				
Key results	18	Summarise key results with reference to study objectives	14–15 & 22	
Limitations	19	Discuss limitations of the study, taking into account sources of potential bias or imprecision. Discuss both direction and magnitude of any potential bias	30	
Interpretation	20	Give a cautious overall interpretation of results considering objectives, limitations, multiplicity of analyses, results from similar studies, and other relevant evidence	26–31	
Generalisability	21	Discuss the generalisability (external validity) of the study results	29–31	
*Other information*				
Funding	22	Give the source of funding and the role of the funders for the present study and, if applicable, for the original study on which the present article is based	33	

*Note*. *Give information separately for cases and controls in case-control studies and, if applicable, for exposed and unexposed groups in cohort and cross-sectional studies.

**Appendix 2:** Table Results of Bonferroni-Corrected Post-Hoc Analyses of Distribution and Differences of Active Aging, Sub-Scales and Total Score, *N* = 1,509.

UJACAS				
Goals	Ability	Opportunity	Activity	Total scale
		Group compared adjusted *p*-value		
*Age*				
55–64 – 65–74 .8	55–64 – 65–74 .9	55–64 – 65–74 1.0	55–64 – 65–74 .003**	55–64 – 65–74 1.0
55–64 – 75–84 .01**	55–64 – 75–84 <.001***	55–64 – 75–84 <.001***	55–64 – 75–84 1.0	55–64 – 75–84 <.001***
55–64 – 85–93 .07	55–64 – 85–93 <.001***	55–64 – 85–93 <.001***	55–64 – 85–93 1.0	55–64 – 85–93 <.001***
65–74 – 75–84 <.001***	65–74 – 75–84 <.001***	65–74 – 75–84 <.001***	65–74 – 75–84 .09	65–74 – 75–84 <.001***
65–74 – 85–93 .01**	65–74 – 85–93 <.001***	65–74 – 85–93 <.001***	65–74 – 85–93 .07	65–74 – 85–93 <.001***
75–84 – 85–93 1.0	75–84 – 85–93 <.001***	75–84 – 85–93***	75–84 – 85–93 .6	75–84 – 85–93 <.001***
*Education*				
Compulsory–Upper secondary (2 years) .5	Compulsory–Upper secondary (2 years) 1.0	Compulsory–Upper secondary (2 years) .5	Compulsory–Upper secondary (2 years) 1.0	Compulsory–Upper secondary (2 years) 1.0
Compulsory–Upper secondary (3–4 years) .001**	Compulsory–Upper secondary (3–4 years) .2	Compulsory–Upper secondary (3–4 years) .06	Compulsory–Upper secondary (3–4 years) 1.0	Compulsory–Upper secondary (3–4 years) .009**
Compulsory–University (up to 3 years) <.001***	Compulsory–University (up to 3 years) .07	Compulsory–University (up to 3 years) .02*	Compulsory–University (up to 3 years) .1	Compulsory–University (up to 3 years) <.001***
Compulsory–University (3 years or longer) <.001***	Compulsory–University (3 years or longer) .009**	Compulsory–University (3 years or longer) <.001***	Compulsory–University (3 years or longer) .03*	Compulsory–University (3 years or longer) <.001***
Upper secondary (2 years)–(Upper secondary school (3–4 years) .8	Upper secondary (2 years)–(Upper secondary school (3–4 years) .3	Upper secondary (2 years)–(Upper secondary school (3–4 years) 1.0	Upper secondary (2 years)–(Upper secondary school (3–4 years) 1.0	Upper secondary (2 years)–(Upper secondary school (3–4 years) .7
Upper secondary (2 years)–University (up to 3 years) .01**	Upper secondary (2 years)–University (up to 3 years) .2	Upper secondary (2 years)–University (up to 3 years) 1.0	Upper secondary (2 years)–University (up to 3 years) .1	Upper secondary (2 years)–University (up to 3 years) .05*
Upper secondary (2 years)–University (3 years or longer) <.001***	Upper secondary (2 years)–University (3 years or longer) .04*	Upper secondary (2 years)–University (3 years or longer) .5	Upper secondary (2 years)–University (3 years or longer) .03*	Upper secondary (2 years)–University (3 years or longer) <.001***
(Upper secondary school (3–4 years)–University (up to 3 years) .9	(Upper secondary school (3–4 years)–University (up to 3 years) 1.0	(Upper secondary school (3–4 years)–University (up to 3 years) 1.0	(Upper secondary school (3–4 years)–University (up to 3 years) 1.0	(Upper secondary school (3–4 years)–University (up to 3 years) 1.0
(Upper secondary school (3–4 years)–University (3 years or longer) .02*	(Upper secondary school (3–4 years)–University (3 years or longer) 1.0	(Upper secondary school (3–4 years)–University (3 years or longer) 1.0	(Upper secondary school (3–4 years)–University (3 years or longer) .6	(Upper secondary school (3–4 years)–University (3 years or longer) .2
University (up to 3 years)–University (3 years or longer) 1.0	University (up to 3 years)–University (3 years or longer) 1.0	University (up to 3 years)–University (3 years or longer) 1.0	University (up to 3 years)–University (3 years or longer) 1.0	University (up to 3 years)–University (3 years or longer) 1.0
*Self-rated health*				
Poor–Fair 1.0	Poor–Fair <.001***	Poor–Fair <.001***	Poor–Fair <.001***	Poor–Fair <.001***
Poor–Good .9	Poor–Good <.001***	Poor–Good <.001***	Poor–Good <.001***	Poor–Good <.001***
Poor–Very good .02*	Poor–Very good* <.001***	Poor–Very good <.001***	Poor–Very good <.001***	Poor–Very good <.001***
Poor–Excellent <.001***	Poor–Excellent <.001***	Poor–Excellent <.001***	Poor–Excellent <.001***	Poor–Excellent <.001***
Fair–Good 1.0	Fair–Good <.001***	Fair–Good <.001***	Fair–Good <.001***	Fair–Good <.001***
Fair–Very good <.001***	Fair–Very good <.001***	Fair–Very good <.001***	Fair–Very good <.001***	Fair–Very good <.001***
Fair–Excellent <.001***	Fair–Excellent <.001***	Fair–Excellent <.001***	Fair–Excellent <.001***	Fair–Excellent <.001***
Good–Very good <.001***	Good–Very good <.001***	Good–Very good <.001***	Good–Very good .01**	Good–Very good <.001***
Good–Excellent <.001***	Good–Excellent <.001***	Good–Excellent <.001***	Good–Excellent <.001***	Good–Excellent <.001***
Very good–Excellent .006**	Very good–Excellent .2	Very good–Excellent <.001***	Very good–Excellent <.001***	Very good–Excellent <.001***

*** p ≤ .001, **≤.01, *≤.05.

## References

[bibr1-30495334251366584] Aljassim N. Ostini R. (2020). Health literacy in rural and urban populations: A systematic review. Patient Education and Counseling, 103(10), 2142–2154. 10.1016/j.pec.2020.06.007 32601042

[bibr2-30495334251366584] Amián J. G. Alarcón D. Fernández-Portero C. Sánchez-Medina J. A. (2021). Aging living at home: Residential satisfaction among active older adults based on the perceived home model. International Journal of Environmental Research and Public Health, 18(17), 8959. 10.3390/ijerph18178959 PMC8431241 34501548

[bibr3-30495334251366584] Baltes M. M. Maas I. Wilms H. U. Borchelt M. Little T. D. (1998). Everyday competence in old and very old age: Theoretical considerations and empirical findings. In Baltes P. B. Mayer K. U. (Eds.), The Berlin aging study (pp. 384–402). Cambridge University Press.

[bibr4-30495334251366584] Boonyaratana Y. Hansson E. E. Granbom M. Schmidt S. M. (2021). The psychometric properties of the meaning of home and housing-related control beliefs scales among 67-70 year-olds in Sweden. International Journal of Environmental Research and Public Health, 18(8), 4273. 10.3390/ijerph18084273 33920612 PMC8072549

[bibr5-30495334251366584] ClinicalTrials.gov. (2021). Prospective RELOC-AGE: How do housing choices and relocation matter for active ageing? (RELOC-AGE). ClinicalTrials.gov. https://clinicaltrials.gov/ct2/show/NCT04765696

[bibr6-30495334251366584] Ekström H. Schmidt S. M. Iwarsson S. (2016). Home and health among different sub-groups of the ageing population: A comparison of two cohorts living in ordinary housing in Sweden. BMC Geriatrics, 16, 90. 10.1186/s12877-016-0265-7 27117314 PMC4847359

[bibr7-30495334251366584] Eronen J. Paakkari L. Portegijs E. Saajanaho M. Rantanen T. (2021). Health literacy supports active aging. Preventive Medicine, 143, 106330. 10.1016/j.ypmed.2020.106330 33220399

[bibr8-30495334251366584] European Commission. (2012). Decision of the European parliament and the Council on the European Year for Active Ageing. European Commission. https://ec.europa.eu/social/main.jsp?langId=en&catId=89&newsId=860

[bibr9-30495334251366584] Fänge A. Iwarsson S. (2003). Accessibility and usability in housing: Construct validity and implications for research and practice. Disability and Rehabilitation, 25(23), 1316–1325. 10.1080/09638280310001616286 14617438

[bibr10-30495334251366584] Fänge A. Iwarsson S. (2005a). Changes in accessibility and usability in housing: An exploration of the housing adaptation process. Occupational Therapy International, 12(1), 44–59. 15962699 10.1002/oti.14

[bibr11-30495334251366584] Fänge A. Iwarsson S. (2005b). Changes in ADL dependence and aspects of usability following housing adaptation—A longitudinal perspective. American Journal of Occupational Therapy, 59, 296–304. 10.5014/ajot.59.3.296 15969277

[bibr12-30495334251366584] Haak M. Kylén M. Ekström H. Schmidt S. M. Horstmann V. Elmståhl S. Iwarsson S. (2015). Relationships between perceived aspects of home and symptoms in a cohort aged 67–70. Archives of Gerontology and Geriatrics, 61(3), 529–534. 10.1016/j.archger.2015.06.013 26199206

[bibr13-30495334251366584] Hobart J. Cano S. (2009). Improving the evaluation of therapeutic interventions in multiple sclerosis: The role of new psychometric methods. Health Technology Assessment, 13(12), iii, ix–x, 1. 10.3310/hta13120 19216837

[bibr14-30495334251366584] Hsu H. C. Liang J. Luh D. L. Chen C. F. Wang Y. W. (2019). Social determinants and disparities in active aging among older Taiwanese. International Journal of Environmental Research and Public Health, 16(16), 3005. 10.3390/ijerph16163005 PMC6721230 31434349

[bibr15-30495334251366584] Inspektionen för socialförsäkringen (ISF). (2017). Kvinnors och mäns pensioner [Women’s and men’s pensions] (Report 2017:08). Inspektionen för socialförsäkringen. https://isf.se/download/18.6e75aae16a591304896bb0/1565330430696/Kvinnors%20och%20ma%CC%88ns%20pensioner-ISF-Rapport%202017-08.pdf

[bibr16-30495334251366584] Iwarsson S. Jönson H. Deierborg T. Ehinger J. K. Hansson O. Isaksson H. Englund M. (2023). ‘Proactive aging’ is a new research approach for a new era. Nature Aging, 3(7), 755–756. 10.1038/s43587-023-00438-6 37291219

[bibr17-30495334251366584] Iwarsson S. Löfqvist C. Oswald F. Slaug B. Schmidt S. Wahl H.-W. Tomsone S. Himmelsbach I. Haak M. (2016). Synthesizing ENABLE-AGE research findings to suggest evidence-based home and health interventions. Journal of Housing for the Elderly, 30(3), 330–343. 10.1080/02763893.2016.1198742

[bibr18-30495334251366584] Kylén M. Schmidt S. M. Iwarsson S. Haak M. Ekström H. (2017). Perceived home is associated with psychological well-being in a cohort aged 67–70 years. Journal of Environmental Psychology, 51, 239–247. 10.1016/j.jenvp.2017.04.006

[bibr19-30495334251366584] Lachman M. E. Neupert S. D. Agrigoroaei S. (2011). Chapter 11. The relevance of control beliefs for health and aging. In Schaie K. W. Willis S. L. (Eds.), Handbook of the psychology of aging (7th ed., pp. 175–190). Academic Press.

[bibr20-30495334251366584] Lawton M. P. Nahemow L. (1973). Ecology and the aging process. In Eisdorfer C. Lawton M. P. (Eds.), The psychology of adult development and aging (pp. 619–674). American Psychological Association.

[bibr21-30495334251366584] Nordeström F. Slaug B. Zingmark M. Granbom M. Rantanen T. Iwarsson S. (2024). Translation and psychometric evaluation of the University of Jyvaskyla Active Aging Scale (UJACAS) for use in Sweden. Journal of Cross-Cultural Gerontology, 39, 17–34. 10.1007/s10823-024-09496-8 38252386 PMC10914907

[bibr22-30495334251366584] Nyqvist F. Nygård M. Snellman F. (2022). Changes in active ageing in a Nordic regional context: Results based on the GERDA study in 2005 and 2016. International Journal of Social Welfare, 31(1), 8–21. 10.1111/ijsw.12480

[bibr23-30495334251366584] Oswald F. Schilling O. Wahl H.-W. Fänge A. Sixsmith J. Iwarsson S. (2006). Homeward bound: Introducing a four-domain model of perceived housing in very old age. Journal of Environmental Psychology, 26(3), 187–201. 10.1016/j.jenvp.2006.07.002

[bibr24-30495334251366584] Oswald F. Wahl H.-W. Martin M. Mollenkopf H. (2003). Toward measuring proactivity in person-environment transactions in late adulthood. Journal of Housing for the Elderly, 17(1-2), 135–152. 10.1300/J081v17n01_10

[bibr25-30495334251366584] Oswald F. Wahl H. W. Schilling O. Iwarsson S. (2007). Housing-related control beliefs and independence in activities of daily living in very old age. Scandinavian Journal of Occupational Therapy, 14(1), 33–43. 10.1080/11038120601151615 17366076

[bibr26-30495334251366584] Oswald F. Wahl H.-W. Schilling O. Nygren C. Fänge A. Sixsmith A. Sixsmith J. Széman Z. Tomsone S. Iwarsson S. (2007). Relationships between housing and healthy aging in very old age. Gerontologist, 47(1), 96–107. 17327545 10.1093/geront/47.1.96

[bibr27-30495334251366584] Rantanen T. Eronen J. Kauppinen M. Kokko K. Sanaslahti S. Kajan N. Portegijs E. (2021). Life-space mobility and active aging as factors underlying quality of life among older people before and during COVID-19 lockdown in Finland - A longitudinal study. Journals of Gerontology. Series A, Biological Sciences and Medical Sciences, 76(3), e60–e67. 10.1093/gerona/glaa274 PMC7665359 33125043

[bibr28-30495334251366584] Rantanen T. Portegijs E. Kokko K. Rantakokko M. Törmäkangas T. Saajanaho M. (2019). Developing an assessment method of active aging: University of Jyvaskyla Active Aging Scale. Journal of Aging and Health, 31(6), 1002–1024. 10.1177/0898264317750449 29291660 PMC6572587

[bibr29-30495334251366584] Roy N. Dubé R. Després C. Freitas A. Légaré F. (2018). Choosing between staying at home or moving: A systematic review of factors influencing housing decisions among frail older adults. PLoS One, 13(1), e0189266. 10.1371/journal.pone.0189266 PMC5749707 29293511

[bibr30-30495334251366584] Siltanen S. Keskinen K. E. Lahti A. M. Rantanen T. von Bonsdorff M. (2024). Active Aging in senior housing residents and community-dwelling older adults: A comparative study in Finland. Journal of Aging and Health, 36, 299–307. 10.1177/08982643231186627 37376762

[bibr31-30495334251366584] Sixsmith J. Sixsmith A. Fänge A. M. Naumann D. Kucsera C. Tomsone S. Haak M. Dahlin-Ivanoff S. Woolrych R. (2014). Healthy ageing and home: The perspectives of very old people in five European countries. Social Science & Medicine, 106, 1–9. 10.1016/j.socscimed.2014.01.006 24524960

[bibr32-30495334251366584] Slaug B. Zingmark M. Granbom M. Björk J. Rantanen T. Schmidt S. M. Iwarsson S. (2024). Meaning of home attenuates the relationship between functional limitations and active aging. Aging Clinical and Experimental Research, 36(1), 159. 10.1007/s40520-024-02810-x 39088106 PMC11294407

[bibr33-30495334251366584] Statistics Sweden. (2022). After age 60. A description of older people in Sweden. (Demographic reports 2022:2). Statistics Sweden (SCB). https://www.scb.se/publication/47310

[bibr34-30495334251366584] Streiner D. Norman G. Cairney J. (2015). Health Measurement Scales: A practical guide to their development and use. Oxford University Press Inc.

[bibr35-30495334251366584] Svendsen M. T. Bak C. K. Sørensen K. Pelikan J. Riddersholm S. J. Skals R. K. Mortensen R. N. Maindal H. T. Bøggild H. Nielsen G. Torp-Pedersen C. (2020). Associations of health literacy with socioeconomic position, health risk behavior, and health status: A large national population-based survey among Danish adults. BMC Public Health, 20(1), 565. 10.1186/s12889-020-08498-8 32345275 PMC7187482

[bibr36-30495334251366584] Tomsone S. Horstmann V. Oswald F. Iwarsson S. (2013). Aspects of housing and perceived health among ADL independent and ADL dependent groups of older people in three national samples. Aging Clinical and Experimental Research, 25(3), 317–328. 10.1007/s40520-013-0050-9 23740591

[bibr37-30495334251366584] UNECE. (2013). Active ageing index 2012. Concept, methodology and final results. https://www.euro.centre.org/downloads/detail/1401

[bibr38-30495334251366584] UNECE. (2018). Active ageing index. Analytical report. https://unece.org/DAM/pau/age/Active_Ageing_Index/ECE-WG-33.pdf

[bibr39-30495334251366584] Wahl H. W. Fange A. Oswald F. Gitlin L. N. Iwarsson S. (2009). The home environment and disability-related outcomes in aging individuals: what is the empirical evidence? Gerontologist, 49(3), 355–367. 10.1093/geront/gnp056 19420315 PMC4854361

[bibr40-30495334251366584] Wahl H. W. Oswald F. (2010). Environmental perspectives on ageing. In Phillipson C. Dannefer D. (Eds.), The Sage handbook of social gerontology (pp. 111–124). Sage Publications Ltd.

[bibr41-30495334251366584] World Health Organization. (2002). Active ageing: A policy framework. World Health Organization. https://apps.who.int/iris/handle/10665/67215

[bibr42-30495334251366584] World Health Organization. (2018). The Global Network for age-friendly cities and communities. World Health Organization. https://www.who.int/publications/i/item/WHO-FWC-ALC-18.4

[bibr43-30495334251366584] Zimmermann J. Hansen S. Wagner M. (2021). Home environment and frailty in very old adults. [Wohnumgebung und Gebrechlichkeit bei hochaltrigen Menschen]. Zeitschrift Fur Gerontologie Und Geriatrie: Organ Der Deutschen Gesellschaft Fur Gerontologie Und Geriatrie, 54(Suppl 2), 114–119. 10.1007/s00391-021-01969-6 PMC8551134 34570266

[bibr44-30495334251366584] Zingmark M. Björk J. Granbom M. Gefenaite G. Nordeström F. Schmidt S. M. Rantanen T. Slaug B. Iwarsson S. (2021). Exploring associations of housing, relocation, and active and healthy aging in Sweden: protocol for a prospective longitudinal mixed methods study. JMIR Research Protocols, 10(9), e31137. 10.2196/31137 PMC8493467 34546172

